# Exploring the training of pharmacists oriented to the demands for clinical pharmacy services: from the perspective of physicians

**DOI:** 10.1186/s12909-023-04353-7

**Published:** 2023-05-22

**Authors:** Hang Jin, Yuankai Huang, Xiaoyu Xi, Lei Chen

**Affiliations:** National Medical Products Administration Key Laboratory for Drug Regulatory Innovation and Evaluation, China Pharmaceutical University, No.639 Longmian Avenue, Jiangning District, Nanjing, Jiangsu Province China

**Keywords:** China, Clinical pharmacists, Physicians, Demands, Perceptions, Expectations, Experiences

## Abstract

**Background:**

To evaluate physicians’ perceptions, experiences and expectations of clinical pharmacists in China from the perspective of physicians’ demands, to improve the training of pharmacists.

**Methods:**

A cross-sectional survey involving physicians (except for primary physicians) was conducted from July to August 2019 in China. Using a field questionnaire, this study gathered data on descriptive information about the respondents and their perceptions, experiences and expectations of clinical pharmacists. Data were analysed descriptively using frequencies, percentages and mean. Several subgroup analyses using Chi-square tests were conducted to identify physicians’ demands for clinical pharmacists in China.

**Results:**

A total of 1376 physicians from secondary and tertiary hospitals in China (response rate = 92%) participated. The majority of the respondents were comfortable with clinical pharmacists providing education to patients (59.09%) and detecting and preventing prescription errors (60.17%), but they appeared uncomfortable when asked about clinical pharmacists suggesting the use of prescription medications to patients (15.71%). Most respondents agreed that clinical pharmacists are a reliable source of general drug information (81.84%) instead of clinical drug information (79.58%). The majority of the respondents expected clinical pharmacists to be knowledgeable drug therapy experts (95.56%) and to educate their patients about the safe and appropriate use of medications (95.56%).

**Conclusion:**

Physicians’ perceptions and experiences were positively associated with the frequency of their interaction with clinical pharmacists. They had high expectations of clinical pharmacists as knowledgeable drug therapy experts. Corresponding policies and measures are needed to improve the education and training system of clinical pharmacists in China.

**Supplementary Information:**

The online version contains supplementary material available at 10.1186/s12909-023-04353-7.

## Background

Pharmaceutical care is defined as direct, responsible, drug-related services provided by pharmacists to the public (including members of the healthcare team, patients, etc.) using pharmacy expertise to improve the patient’s quality of life [1, 2]. It has been increasingly valued for its significant clinical outcomes and economic benefits [3, 4]. In clinical practice, physicians directly influence the practice of clinical pharmacy services (CPSs) [5, 6]. Clinical pharmacists act as pharmacotherapy experts within the healthcare team, providing the team with the required expertise [7]. Clarifying physicians’ demands for clinical pharmacists will help foster physicians’ support and confidence in clinical pharmacists and further develop their collaborative relationship [8, 9]. Investigating physicians’ perceptions, experiences and expectations of the role of clinical pharmacists can provide a comprehensive understanding of physicians’ demands for clinical pharmacists [10–15]. Perception – the degree of comfort with clinical pharmacists’ support of physicians’ treatment; experience – the acceptance or assessment of CPS provided by clinical pharmacists; and expectation – the ability and competence of clinical pharmacists to enhance patient safety and support physicians’ decision-making [16]. Compared to developed countries, the roles and work patterns of clinical pharmacists in developing countries, including China, are still less defined [17], and the training mode of clinical pharmacy talents is in the exploratory stage [18]. Understanding physicians’ demands and expectations of clinical pharmacists’ role in the health services delivery can help to inform the training of future clinical pharmacist.

Based on empirical studies conducted around the globe, the demands of clinical pharmacists may vary between physicians with different characteristics, such as clinical speciality [19]. One of the main reasons why clinical pharmacists can play an effective role in collaborative practice with other members of the healthcare team is their ability to integrate basic pharmaceutical science with other clinical expertise [20]. As physicians in different specialities are responsible for managing different diseases, the demands for clinical pharmacists may vary from one clinical speciality to another. Therefore, conducting subgroup analysis in the survey analysis to distinguish between the demands of clinical pharmacists by physicians with different characteristics may lead to supplementary findings.

Compared to developed countries, the development of clinical pharmacy in China is still in the primary phase [21]. The Provisions on the Administration of Pharmaceutical Affairs in Medical Institutions require that medical institutions should have an appropriate number of clinical pharmacists according to the nature and scale of the institution, with no less than five clinical pharmacists in tertiary hospitals and no less than three clinical pharmacists in secondary hospitals. Clinical pharmacists should be involved full-time in clinical drug therapy, educating patients on the use of medication and guiding them in the safe use of medication. However, many Chinese hospitals are unable to meet this requirement. One of the reasons for the slow development of clinical pharmacy in China is the lack of clinical pharmacists and the inadequacy of the pharmaceutical care capacity of pharmacists [22]. Therefore, strengthening clinical pharmacy education in China is an urgent issue at present [23]. The training of clinical pharmacy talents in China is mainly based on undergraduate education and master’s degree education in clinical pharmacy [24]. Although the scale of pharmacy education in China is developing rapidly, there are certain problems: the lack of curriculum subjects that integrate pharmacy and medicine, resulting in a disconnect in the knowledge that does not well meet the demands of physicians [25]. Studies have shown that most interviewed physicians questioned the competence of clinical pharmacists [26, 27], believing that the current general business competence of clinical pharmacists is generally low [28] and that the pharmacy background of clinical pharmacists does not meet the clinical needs [29]. Education should primarily serve the needs of social development, and investigating the demands of physicians for clinical pharmacists may be one of the main entry points for improving the training program of clinical pharmacy personnel in China [30].

The demands of physicians for clinical pharmacists in China is not clear. There is a lack of studies investigating physicians’ demands for clinical pharmacists in terms of perceptions, expectations, and experiences in China. Most of the studies focused on the theoretical discussions of the clinical pharmacists’ responsibilities and skill sets needed by the physicians without an in-depth analysis of physicians’ demands for clinical pharmacists [31, 32]. Some studies have limitations on the timeliness of data, sample sizes and representativeness of the sample and no subgroup analysis was carried out [33, 34], and thus have little reference value for improving the training of clinical pharmacists in China. Based on the analysis above, we investigated Chinese physicians’ perceptions, expectations and experiences of clinical pharmacists from the perspective of physicians’ demands. The findings of this study can be used to inform the training requirement of clinical pharmacists in China. This study may also provide valuable insights into clinical pharmacy education in developing countries.

## Methods

### Study design and participants

Hospitals in China can be divided into primary, secondary and tertiary healthcare institutions based on their scale, research direction, technical talent and medical hardware equipment. As stipulated in the relevant regulations, the health authority formulates plans for the development of CPS only in secondary and tertiary hospitals, and primary healthcare institutions have not been equipped with clinical pharmacists until recently. Consequently, this study investigates the demands of physicians regarding the role of clinical pharmacists only in secondary and tertiary hospitals.

This study applied a stratified sampling strategy. The sample covered all 31 provincial administrative regions (including provinces, autonomous regions, and municipalities directly under the central government) in mainland China. Three provinces (autonomous regions) were excluded from the sample due to the difficulty in data collection. First, all the counties in each individual provincial administrative unit were divided into three groups based on the local gross domestic product per capita of 2018. Second, the study randomly selected one county within each group for a total of 93 counties. Third, at least four tertiary hospitals and four secondary hospitals in each sampled county were selected according to the accessibility of their information and the convenience of the actual survey. Fourth, within each of the sample hospitals, at least two full-time physicians were selected as respondents by the convenience sampling method. Visiting scholars, trained physicians, trainees and interns at the hospital were excluded from the sample. It is expected that 93*8*2 = 1488 physicians will be invited to participate in the study.

### Instruments

The questionnaire used in this study was adapted from previously published studies with minor modifications to make it applicable to China [11]. The questionnaire consisted of four sections to collect descriptive information about the respondents and to measure their perceptions, experiences and expectations of clinical pharmacists.

Section 1 collected the respondents’ socio-demographic and work-related information. Previous studies have suggested that factors associated with physicians’ perceptions, experiences and expectations include socio-demographic factors (age, gender, education level) and work-related factors (current setting of practice, length of service, professional title, executive position, type of hospital, geographic position of hospital, and frequency of collaboration between physicians and clinical pharmacists) [10, 13, 14, 19, 35–38].

Section 2 assessed the perceptions of different CPSs provided by clinical pharmacists. The respondent’s degree of comfort with different CPSs was assessed using a 3-point Likert scale from 1 (uncomfortable) to 3 (comfortable).

Sections 3 and 4 assessed the expectations of physicians and their experiences with clinical pharmacists, respectively. Each part of the questionnaire was an 8-item scale scored on a 5-point Likert scale. The scale anchors ranged from 1 (strongly disagree) to 5 (strongly disagree).

To adapt the questionnaire from the English version to the local context and to ensure the accuracy of the translation, two translators with adequate knowledge of pharmaceutical care were recruited. One translator was a native Chinese speaker who was proficient in English, and the other was a native English speaker who was proficient in Chinese. The two translators independently translated the items and responses, and the translations were then proofread and revised until the two translators finalized their versions. Then, five physicians from tertiary hospitals and three physicians from secondary hospitals were invited to compare and discuss the two translations, and minor adjustments were made to improve the clarity of some of the items. Thus, a formal Chinese version of the questionnaire for the study was developed.

To improve clarity and reduce response bias, the questionnaire was pre-tested among fifteen physicians before the study. The results showed that the questionnaire was reasonable, understandable and readable. the reliability and validity of the questionnaire measuring the physicians’ perceptions, experience and expectations of this study are acceptable (Table 1).

**Table 1:** Validity and Reliability of Questionnaire

		Perceptions	Experiences	Expectations
The KMO measurement of sampling adequacy		0.527	0. 543	0. 711
Bartlett’s Test of Sphericity	Approx. Chi-square	307. 439	401.679	421. 836
	df	7	7	7
	Sig.	0.00	0.00	0.00
Cronbach’s alpha		0.75	0.79	0.69
N of items		8.00	8.00	8.00

### Data collection

Sixteen data collection assistants were appointed to investigate in each provincial administrative region. A total of 496 data collection assistants with a higher education background in pharmacy or pharmaceutical science were recruited for data collection. All data collection assistants were well trained in the background, purpose, and methods of our survey by our research team and appropriate professionals. The survey was conducted in July and August 2019. An informed consent form was provided to the director of each sampled hospital and the physicians involved in the survey, and the survey was conducted after acquiring written informed consent from each participant. To ensure the validity and quality of the data, the data collection assistants informed potential respondents of the purpose and contents of this survey during their nonwork time. Then, the questionnaires were administered with survey software through face-to-face interviews.

### Data analysis

The descriptive analysis was conducted to determine the respondents’ characteristics and their responses to the questionnaire items: frequency (%) for categorical data and mean (SD) for continuous data. Several subgroup analyses were conducted in this study, using Chi-square tests to test the significance of association between the independent variables (gender, age, current setting of practice, length of service, educational background, type of hospital, geographic position, frequency of interaction) and the dependent variables (respondent’s level of comfort, expectations and experiences). The results were considered significant at a *p-value* of < 0.05. SPSS 24 (IBM Corporation, Armonk, NY, USA) was used to analyze the data.

## Results

### Demographics

The survey collected 1376 valid questionnaires from physicians, with a response rate of 92%. The final valid sample contained a total of 1376 respondents (Table 2). The mean (SD) age of the respondents was 37.80 (9.26) years. The mean (SD) length of service was 13.16 (11.95) years. Males predominated in the physician sample at 53.05%. Among the respondents, 26.89% of them worked in internal medicine departments, which is a greater number than in any other department. The results showed that the sample was evenly distributed.

**Table 2:** Demographics and Relevant Characteristics of Participants

Characteristics	No. (%) Physicians(*n* = 1376)
Gender	
Male	730 (53.05)
Female	646 (46.95)
Age, Mean (SD)	37.80 (9.26)
The current setting of practice	
Internal medicine	370 (26.89)
General surgery	199 (14.46)
Obstetrics, gynecology & pediatrics	170 (12.35)
Oncology	34 (2.47)
Emergency	45 (3.27)
Others	558 (40.55)
Length of serving, Mean (SD)	13.16 (11.95)
Professional title	
Junior	476 (34.59)
Intermediate	557 (40.48)
Vice-senior	265 (19.26)
Senior	78 (5.67)
Executive positions	
Director of the section	147 (10.68)
Vice director of section	172 (12.50)
General physician	1057 (76.82)
Education level	
Below undergraduate degree	123 (8.94)
Bachelor’s degree	894 (64.97)
Master’s degree	310 (22.53)
Doctoral degree	49 (3.56)
Types of hospital	
Tertiary hospital	730 (53.1)
Secondary hospital	646 (46.9)
Geographic position	
Eastern	506 (36.8)
Center	490 (35.6)
Western	380 (27.6)
Frequency of collaboration	
Never or rarely	576 (41.86)
Once a week or more	653 (47.46)
Once a day or more	147 (10.68)
Reasons for interactions (multiple-choice)	
Drug availability queries	665 (48.33)
Drug alternatives queries	681 (49.49)
Drug dosage queries	678 (49.27)
Side effects queries	728 (52.91)
Drug interactions queries	678 (47.27)
Other	340 (24.71)

Physician-clinical pharmacist interactions were not very frequent. Of the physician respondents, 41.86% declared that they never or rarely interacted with clinical pharmacists, less than half of respondents (47.46%) interacted with clinical pharmacists at least once a week, while only 147 (10.68%) interacted daily with clinical pharmacists.

### Perceptions of physicians

Table 3 shows the respondents’ perceptions of clinical pharmacists providing different CPSs. The majority of the respondents were comfortable with clinical pharmacists detecting and preventing prescription errors (60.17%) and providing education to patients (59.09%). However, a small number of the respondents were uncomfortable with clinical pharmacists suggesting the use of nonprescription medications (12.74%) or prescription medications (15.71%) to patients or treating minor illnesses (10.50%). Appendix A shows the subgroup analyses of respondents’ perceptions of clinical pharmacists. There was a significant correlation between the frequency of interactions and physicians’ perceptions of clinical pharmacists. Regarding this association, the respondents who had daily interactions with clinical pharmacists were more comfortable than those who interacted less frequently. There were no significant associations with variables other than those listed in Appendix A.

**Table 3:** Physicians’ Perceptions of Clinical Pharmacists

Item	No. (%)			Mean (SD)
	Uncomfortable	Moderately comfortable	Comfortable	
[1] Providing patient education	61 (4.42)	504 (36.50)	816 (59.09)	2.55 (0.580)
[2] Suggesting use of non-prescription medications to patients, e.g. paracetamol	176 (12.74)	582 (42.14)	623 (45.11)	2.32 (0.689)
[3] Suggesting use of prescription medications to patients, e.g. antibiotics	217 (15.71)	541 (39.17)	623 (45.11)	2.29 (0.723)
[4] Suggesting use of prescription medications to physicians	92 (6.66)	571 (41.35)	718 (51.99)	2.45 (0.618)
[5] Treating minor illnesses, e.g. headaches	145 (10.50)	586 (42.43)	650 (47.07)	2.37 (0.665)
[6] Designing and monitoring pharmacotherapeutic regimes	82 (5.94)	561 (40.62)	738 (53.44)	2.48 (0.607)
[7] Monitoring outcomes of pharmacotherapeutic regimens	63 (4.56)	563 (40.77)	755 (54.67)	2.50 (0.584)
[8] Detecting and preventing prescription errors	70 (5.07)	480 (34.76)	831 (60.17)	2.55 (0.591)

### Experiences of physicians

The physicians’ actual experiences with clinical pharmacists is shown in Table 4. A large percentage of the respondents agreed or strongly agreed that clinical pharmacists were a reliable source of general drug information (81.84%) as opposed to clinical drug information (79.58%). Approximately four-fifths of the respondents agreed or strongly agreed that clinical pharmacists routinely informed them if they discovered clinical problems with their prescriptions (82.63%). The respondents strongly disagreed or disagreed that clinical pharmacists routinely informed them about more cost-effective alternatives to the drugs they prescribed (10.61%) and took personal responsibility for resolving any drug-related problems they discovered (13.3%).

**Table 4:** Physicians’ Experience with Clinical Pharmacists

Physicians’ experience	Participant response, No. (%)					Mean (SD)
	Strongly disagree	Disagree	Neutral	Agree	Strongly agree	
[9] In my experience, clinical pharmacists are a reliable source of general drug information (e.g., specific facts about drugs which can be found in standard references)	4 (0.30)	56 (4.10)	190 (13.80)	896 (64.90)	230 (16.70)	3.94 (0.701)
[10] In my experience, clinical pharmacists are a reliable source of clinical drug information (e.g., information regarding the clinical use of drugs in specific situations)	4 (0.30)	57 (4.10)	220 (15.90)	883 (63.90)	212 (15.40)	3.90 (0.705)
[11] Clinical pharmacists routinely counsel my patients regarding the safe and appropriate use of their medications	11 (0.80)	65 (4.70)	218 (15.80)	845 (61.20)	237 (17.20)	3.90 (0.762)
[12] Clinical pharmacists routinely inform me if they discover clinical problems with my prescriptions’	5 (0.40)	62 (4.50)	172 (12.50)	898 (65.00)	239 (17.30)	3.95 (0.714)
[13] Clinical pharmacists routinely inform me about more cost-effective alternatives to the drugs I prescribe	15 (1.10)	131 (9.50)	293 (21.20)	747 (54.10)	190 (13.80)	3.70 (0.861)
[14] Clinical pharmacists frequently ask me to clarify for them the drug therapy objectives I have in mind for my patients	12 (0.90)	108 (7.80)	218 (15.80)	846 (61.30)	192 (13.90)	3.80 (0.806)
[15] Clinical pharmacists frequently let me know that my patients have experienced some problem with their medication	8 (0.60)	90 (6.50)	189 (13.70)	875 (63.40)	214 (15.50)	3.87 (0.768)
[16] In my experience, clinical pharmacists appear willing to take personal responsibility for resolving any drug-related problems they discover	27 (2.00)	156 (11.30)	415 (30.10)	642 (46.50)	136 (9.80)	3.51 (0.890)

Appendix B shows the subgroup analyses in respondents’ experiences of clinical pharmacists. All the variables significantly correlated with each item of the experience questionnaire are listed in Appendix B. The geographic position of physicians was related to significant differences in the following three items: (1) pharmacists are a reliable source of clinical drug information (*p =* 0.019); (2) pharmacists routinely inform physicians about more cost-effective alternatives to the drugs they prescribe (*p* = 0.019); and (3) pharmacists appear willing to take personal responsibility for resolving any drug-related problems they discover (*p* = 0.049). Respondents with junior titles were more likely to agree or strongly agree that their experiences with clinical pharmacists indicated that pharmacists (1) are a reliable source of general drug information (*p* = 0.021); (2) inform physicians if they discover clinical problems with their prescriptions (*p* = 0.031); (3) inform physicians about more cost-effective alternatives to the drugs they prescribe (*p* = 0.001); (4) ask physicians to clarify drug therapy objectives for their patients (*p* = 0.000); and (5) let physicians know if patients experience problems with their medications (*p* = 0.011). Additionally, there was a significant difference with respect to their level of contact with pharmacists. Frequently interacting respondents were more likely to have positive experiences with clinical pharmacists.

### Expectations of physicians

The results showing physicians’ expectations of the roles of clinical pharmacists are listed in Table 5. A large percentage of the respondents agreed or strongly agreed that clinical pharmacists are expected to (1) be knowledgeable drug therapy experts (95.56%); (2) educate their patients about the safe and appropriate use of medication (95.56%); (3) monitor their patients’ response to drug therapy and let them know if a patient encounters any drug-related problem (92.52%); and (4) know the specific indications of each drug they prescribe, even when the drugs have more than one approved or recognized indication (90.04%). These findings suggest that Chinese clinical pharmacists should improve their work in these four aspects.

**Table 5:** Physicians’ Expectation of Clinical Pharmacists

Item	Participant response, No. (%)					Mean (SD)
	Strongly disagree	Disagree	Neutral	Agree	Strongly agree	
[17] I expect clinical pharmacists to take personal responsibility for resolving any drug-related problems they discover involving patients	23 (1.70)	202 (14.60)	204 (14.80)	740 (53.60)	207 (15.00)	3.66 (0.959)
[18] I expect clinical pharmacists to be knowledgeable drug therapy experts	16 (1.20)	6 (0.40)	39 (2.80)	779 (56.40)	536 (38.80)	4.32 (0.663)
[19] I expect clinical pharmacists to assist me in designing drug therapy treatment plans for my patients	14 (1.00)	39 (2.80)	107 (7.70)	885 (64.10)	331 (24.00)	4.08 (0.720)
[20] I expect clinical pharmacists to educate my patients about the safe and appropriate use of their medication	12 (0.90)	15 (1.10)	52 (3.80)	870 (63.00)	427 (30.90)	4.22 (0.648)
[21] I expect clinical pharmacists to monitor my patients’ response to drug therapy and let me know if a patient encounters any drug-related problem	9 (0.70)	14 (1.00)	80 (5.80)	892 (64.60)	381 (27.60)	4.18 (0.635)
[22] I expect clinical pharmacists to know the specific indication of each drug I prescribe, even when drugs have more than one approved or recognized indication	12 (0.90)	15 (1.10)	110 (8.00)	906 (65.60)	333 (24.10)	4.11 (0.657)
[23] I expect clinical pharmacists to be available to me for consultation when I see patients (e.g. during rounds)	14 (1.00)	50 (3.60)	149 (10.80)	859 (62.20)	304 (22.00)	4.01 (0.753)
[24] I expect clinical pharmacists to assist my patients in selecting appropriate non-prescription medications	10 (0.70)	67 (4.90)	136 (9.80)	882 (63.90)	281 (20.30)	3.99 (0.751)

Appendix C shows the subgroup analyses in respondents’ expectations of clinical pharmacists. All the variables significantly correlated with each item of the expectation questionnaire are listed in Appendix C. The level of expectations was higher among physicians from tertiary hospitals than those from secondary hospitals. In particular, the differences were statistically significant for three items: (1) I expect clinical pharmacists to assist me in designing drug therapy treatment plans for my patients (*p* = 0.041); (2) I expect clinical pharmacists to educate my patients about the safe and appropriate use of their medication (*p* = 0.008); and (3) I expect clinical pharmacists to know the specific indication of each drug I prescribe, even when the drugs have more than one approved or recognized indication (*p* = 0.031). A significant association was observed between the responses and the current practice setting. Oncologists expected clinical pharmacists to improve their knowledge of the drug therapy (*p* = 0.002), monitor patients’ response to drug therapy and let the physician know if a patient encountered any drug-related problems in relation to knowledge and skills of tumour treatment and his/her use of drugs with different disease progressions(*p* = 0.004). Emergency room physicians expected clinical pharmacists to assist them in quickly designing drug therapy treatment plans (*p* = 0.009).

## Discussion

This is the first large-scale survey of physicians’ demands for clinical pharmacists in China. The physicians’ perceptions, experiences, and expectations regarding the role of clinical pharmacists were quantitatively analyzed. On this basis, subgroup analysis was used to examine differences in the demands for clinical pharmacists among physicians with different socio-demographic factors and different work-related factors. The results revealed that the demands of physicians for Chinese clinical pharmacists are not well met and there are significant differences in the perceptions and experiences of clinical pharmacists among physicians who collaborate with them at different frequencies. In addition, there were significant differences in the experience of clinical pharmacists among physicians from different regions and professional titles, as well as in the expectations of clinical pharmacists among physicians from different levels of hospitals and departments.

In China, the reported frequency of physicians’ interactions with clinical pharmacists is low. Only 11% of physicians engaged in communication with clinical pharmacists at least once a day, while more than 40% of physicians never or rarely interacted with clinical pharmacists (Table 2). Interaction between physicians and clinical pharmacists is infrequent due to the lack of opportunities for clinical pharmacists to communicate effectively with physicians and the neglect of the development of communication skills for students in medical schools [39]. Interprofessional education (IPE) has been recognized internationally as a way to improve healthcare professional interactions [40]. At present, clinical pharmacy talents in China mainly come from pharmacy majors in higher education. Only some of these universities offer pharmacy practice education, and the collaboration and interaction between pharmacy and other disciplines are not fully reflected in the practice teaching process. Therefore, to improve the collaboration between clinical pharmacists and healthcare team members, IPE should be introduced into the training of clinical pharmacy talents, break the professional barriers and establish a multi-dimensional and multi-directional inter-professional cooperative education platform.

Respondents in this study were asked about their perceptions of clinical pharmacists providing different CPSs (Table 3). Physicians were comfortable with the patient-oriented CPSs provided by clinical pharmacists. However, some physicians appeared uncomfortable with clinical pharmacists prescribing, such as suggesting prescription medications to patients and treating patients with minor illnesses. This supports previous studies, which demonstrated that these direct interactions with patients make physicians uncomfortable [19, 36]. There could be several reasons for this discomfort. First, physicians are unsure whether clinical pharmacists are competent to independently prescribe medications. Second, physicians believe that the right to prescribe belongs to them. Clinical pharmacists prescribing medication could intrude into the physicians’ realm and threaten the medical dominance of physicians. Third, physicians may feel that clinical pharmacists prescribing medications would damage the physician-patient relationship if the clinical pharmacists’ suggestions differ from their own [41]. To avoid such conflicts, the authority of clinical pharmacists to prescribe should be legally granted, as it has in the United States [42], United Kingdom [43] and Canada [44]. At the same time, compared to pharmacist prescribing competency education abroad, no specific pharmacist prescribing courses have been developed in China, and the requirements for core prescribing skills such as diagnosis are not high. We can follow the example of New Zealand in providing standardized prescribing training, with training courses on core prescribing skills such as diagnostic, clinical reasoning and assessment skills, to improve the pharmacy service capacity of prescribers [45].

In the survey of the physicians’ experience with clinical pharmacists (Table 4), the respondents strongly disagreed or disagreed that clinical pharmacists routinely informed them about more cost-effective alternatives to the drugs they prescribed (10.61%) and took personal responsibility for resolving any drug-related problems they discovered (13.3%). For the former, cost-effective alternatives are drugs that are selected by clinical pharmacists for physicians based on pharmacoeconomic evaluations for their positive efficacy, low adverse effects, reasonable price and ease of use. This is consistent with a study which concluded that most low- and middle-income countries face a shortage of qualified clinical pharmacists in proposing cost-effective alternatives and how to address these issues in practice [38]. Therefore, the training of clinical pharmacists in pharmacoeconomics should be strengthened so that clinical pharmacists can apply the principles and methods of economics in practice to find cost-effective treatment options for the healthcare team in order to achieve the maximum use of health resources [46]. For the latter, although the relevant provisions require them to participate in clinical consultation, there is no clear definition of their qualifications, rights, and the division of responsibilities in the event of disputes. The involvement of clinical pharmacists in clinical drug therapy entails more workload and responsibilities, and naturally the risks they face are increasing, thus making them susceptible to physicians’ doubts. [47].

According to Chi-square tests, in the subgroup analyses of respondents’ experiences with clinical pharmacists, findings show that the geographic position of physicians was related to significant differences. The respondents in the eastern region were more satisfied with the clinical drug information provided by clinical pharmacists, which was closely related to the higher professional quality and level of clinical pharmacists in the eastern region. In addition, respondents with junior titles were more likely to agree or strongly agree their experiences with clinical pharmacists. The possible reason for this is that physicians with junior title need more support from clinical pharmacists in terms of pharmacotherapy expertise than physicians with higher titles, due to their insufficient clinical work experience and theoretical knowledge.

Overall, the frequency of communication between physicians and clinical pharmacists on routine matters exerts a positive influence on physicians’ perceptions and experience of CPS. This finding emphasizes that physicians who interact more positively and frequently with clinical pharmacists recognize the benefits that clinical pharmacists offer through their work, such as providing physicians with valuable suggestions concerning pharmacotherapy and decreasing their workload. To obtain the mutual benefit of collaboration, clinical pharmacists should therefore be proactive in the interactions with physicians. For example, clinical pharmacists can provide physicians with suggestions on dosage accuracy, the appropriate duration of pharmacotherapy and the rational choice of drugs to prove their capability and make a positive impression.

In the subgroup analyses of respondents’ expectations of clinical pharmacists, types of hospitals and the current setting of practice were significantly associated with respondents’ expectations. Physicians in hospitals with higher grades have higher expectations for clinical pharmacists to provide a variety of CPSs. The possible explanation is that in China, hospitals with higher grades provide health care for patients with more complicated diseases and bear a greater burden of health care, which places greater demands on CPSs to resolve the issue associated with the therapeutic management of a disease including adverse effects and complications arising from medication use. In addition, there are differences in the CPSs needed by different departments. Oncologists expected clinical pharmacists to have more knowledge of drug therapy, while emergency room physicians expected clinical pharmacists to assist them in quickly designing drug therapy treatment plans. This result is related to the characteristics of the disease treatment undertaken by the department.

Regarding physicians’ previous experience, the results showed that 80% of the physicians agreed that clinical pharmacists are a reliable source of general drug information (Table 4) and clinical drug information, yet approximately 96% expected clinical pharmacists to be knowledgeable pharmacotherapy experts (Table 5). These results were similar to several previously published studies [11, 19, 37, 38, 48]. This finding indicates that clinical pharmacists are less competent to provide certain information to meet the actual needs of physicians, possibly due to clinical pharmacists’ lack of knowledge of pharmacotherapy. Some clinical pharmacists have not obtained a degree in pharmacy or clinical pharmacy and thus have not studied the clinical content of the pharmacy curriculum or received adequate clinical pharmacy training [49, 50]. Thus, higher recruitment standards and more training programs for urgently needed knowledge and skills may improve clinical pharmacists’ professional level. The American College of Clinical Pharmacy (ACCP) has developed a coherent system of multiyear postgraduate training [51] to enhance and reinforce clinical pharmacists’ competencies, which is worth considering in China. Nevertheless, the coexistence of multiple training mechanisms has led to much debate about standards of care and affected the quality and efficiency of pharmaceutical care in China. Considering the needs of different types of physicians, the health authority should establish appropriate and systematic training systems and offer continuing education programs in pharmacotherapy for clinical pharmacists. There is a gap between the expectations of Chinese physicians and the actual capabilities of clinical pharmacists, and clinical pharmacists face a huge demand for professional learning. Continuing Professional Development (CPD) is defined as an ongoing, self-directed, structured, and outcomes-focused learning cycle focused on maintaining and improving performance of professional practice [52]. CPD is encouraged to be explored and implemented in a number of countries, including the UK, Canada, New Zealand and the USA, to enhance continuing education for pharmacists [53]. Addressing the needs of physicians and pharmacists through targeted work-based CPD is more likely to support pharmacists in their transition to new roles, such as situated learning in the workplace [54]. Chinese hospital pharmacy faces an urgent need to improve the capacity and quality of clinical pharmacy services [55]. Relevant government departments and hospital pharmacy departments should actively explore the practical application of continuous professional development in China’s healthcare delivery system and further enhance the comprehensive level of clinical pharmacists through continuing pharmacy education so as to meet the needs of pharmacy service transformation.This study has some limitations. First, the representativeness of prefecture-level city hospitals of the entire Chinese public hospital system is valid only in relation to key elements of CPS. Due to significant differences in size, capability, function, and other aspects of public and private hospitals, the illustration of the whole picture requires a series of studies focusing on specific types of hospitals. Second, some issues derived from the discussion require additional data and references for further discussion and precise conclusions. For example, the reasons that physicians appeared uncomfortable with clinical pharmacists prescribing and all assumptions mentioned in the discussion are based merely on the limited data of our survey, the information provided in other research, or our knowledge of the current situation but without solid supporting data.

## Conclusion

Clinical pharmacists’ functions are limited to their traditional roles, leaving few healthcare human resources to develop their role in direct patient care in partnership with physicians. Physicians are reluctant to accept the role of clinical pharmacists in prescribing medications, even for minor illnesses. Physicians’ perceptions and experiences of clinical pharmacists are positively associated with the frequency of their interaction with clinical pharmacists. Physicians have high expectations of clinical pharmacists as knowledgeable drug therapy experts, but at the moment, clinical pharmacists are unable to perform this role in China. Specific efforts should be made to improve clinical pharmacists’ competence, increase physicians’ acceptance and shape a better operative environment for clinical pharmacists.

## Electronic supplementary material

Below is the link to the electronic supplementary material.

Supplementary Material 1

## Data Availability

The datasets used and/or analysed during the current study are available from the corresponding author on reasonable request.

## Abbreviations

CPS: Clinical pharmacy service
