# Supplementary material for: Exploring the training of pharmacists oriented to the demands for clinical pharmacy services: from the perspective of physicians
Source: BMC Med Educ. 2023 May 22;23:357. doi: 10.1186/s12909-023-04353-7 (PMC10201797; doi:10.1186/s12909-023-04353-7)
Supplement: Supplementary file 1 — Supplementary Material 1 [file 12909_2023_4353_MOESM1_ESM.docx]

**Appendix A. Correlation of Variables with Stated Items in Perceptions Questionnaire**

| **Variables** | **M (SD)** | | | | | | | |
| --- | --- | --- | --- | --- | --- | --- | --- | --- |
|  | **[1]** | **[2]** | **[3]** | **[4]** | **[5]** | **[6]** | **[7]** | **[8]** |
| **Frequency of Collaboration** | | | | | | | | |
| Never or rarely | 2.52 (0.604) | 2.30 (0.700) | 2.27 (0.726) | 2.42 (0.628) | 2.35 (0.675) | 2.44 (0.607) | 2.47 (0.595) | 2.52 (0.604) |
| Once a week or more | 2.56 (0.566) | 2.34 (0.685) | 2.31 (0.710) | 2.47 (0.610) | 2.37 (0.664) | 2.50 (0.606) | 2.52 (0.574) | 2.56 (0.566) |
| Once a day or more | 2.59 (0.546) | 2.33 (0.666) | 2.30 (0.771) | 2.48 (0.612) | 2.42 (0.629) | 2.49 (0.612) | 2.52 (0.589) | 2.59 (0.546) |
| *p value* | 0.000*** | 0.001** | 0.001** | 0.002** | 0.003** | 0.022* | 0.006** | 0.025* |

** p*< 0.05

*** p*< 0.01

**** p*< 0.001

**Appendix B. Correlation of Variables with Stated Items in Experience Questionnaire**

| **Variables** | **M (SD)** | | | | | | | |
| --- | --- | --- | --- | --- | --- | --- | --- | --- |
|  | **[9]** | **[10]** | **[11]** | **[12]** | **[13]** | **[14]** | **[15]** | **[16]** |
| Geographic Position | | | | | | | | |
| Eastern | 3.97 (0.748) | 3.90 (0.780) | 3.90 (0.751) | 3.95 (0.917) | 3.65 (0.870) | 3.75 (0.828) | 3.85 (0.915) | 3.97 (0.748) |
| Center | 3.89 (0.715) | 3.83 (0.729) | 3.88 (0.678) | 3.91 (0.862) | 3.66 (0.775) | 3.83 (0.796) | 3.84 (0.885) | 3.89 (0.715) |
| Western | 3.95 (0.643) | 3.97 (0.770) | 3.91 (0.702) | 3.97 (0.793) | 3.79 (0.760) | 3.82 (0.678) | 3.91 (0.865) | 3.95 (0.643) |
| *p value* | 0.281 | 0.019* | 0.810 | 0.466 | 0.019* | 0.284 | 0.386 | 0.049* |
| Professional Title | | | | | | | | |
| Junior | 3.99 (0.677) | 3.95 (0.744) | 3.93 (0.677) | 3.97 (0.806) | 3.80 (0.769) | 3.89 (0.714) | 3.93 (0.888) | 3.99 (0.677) |
| Intermediate | 3.95 (0.669) | 3.92 (0.748) | 3.92 (0.695) | 3.98 (0.854) | 3.69 (0.780) | 3.82 (0.739) | 3.89 (0.887) | 3.95 (0.669) |
| Vice-senior | 3.88 (0.787) | 3.83 (0.791) | 3.80 (0.762) | 3.83 (0.921) | 3.54 (0.864) | 3.64 (0.842) | 3.77 (0.887) | 3.88 (0.787) |
| Senior | 3.77 (0.800) | 3.78 (0.858) | 3.87 (0.852) | 3.97 (0.956) | 3.76 (0.921) | 3.67 (0.968) | 3.71 (0.935) | 3.77 (0.800) |
| *p value* | 0.021* | 0.066 | 0.113 | 0.031* | 0.001** | 0.000*** | 0.011* | 0.367 |
| Frequency of Collaboration | | | | | | | | |
| Never or rarely | 3.85 (0.747) | 3.76 (0.769) | 3.76 (0.800) | 3.79 (0.777) | 3.59 (0.894) | 3.67 (0.854) | 3.70 (0.869) | 3.38 (0.931) |
| Once a week or more | 3.99 (0.649) | 3.98 (0.628) | 3.96 (0.727) | 4.04 (0.635) | 3.78 (0.807) | 3.88 (0.744) | 3.98 (0.667) | 3.58 (0.831) |
| Once a day or more | 4.07 (0.693) | 4.10 (0.666) | 4.13 (0.665) | 4.14 (0.669) | 3.81 (0.917) | 3.92 (0.815) | 4.03 (0.640) | 3.70 (0.917) |
| *p value* | 0.000*** | 0.000*** | 0.000*** | 0.000*** | 0.000*** | 0.000*** | 0.000*** | 0.000*** |

** p*< 0.05

*** p*< 0.01

**** p*< 0.001

**Appendix C. Correlation of Variables with Stated Items in Expectation Questionnaire**

| **Variables** | **M (SD)** | | | | | | | |
| --- | --- | --- | --- | --- | --- | --- | --- | --- |
|  | **[17]** | **[18]** | **[19]** | **[20]** | **[21]** | **[22]** | **[23]** | **[24]** |
| Types of Hospital | | | | | | | | |
| Tertiary hospital | 3.65 (0.965) | 4.3 (0.672) | 4.04 (0.760) | 4.18 (0.667) | 4.15 (0.652) | 4.08 (0.661) | 4.01 (0.758) | 3.98 (0.777) |
| Secondary hospital | 3.67 (0.954) | 4.34 (0.653) | 4.12 (0.669) | 4.27 (0.623) | 4.21 (0.615) | 4.15 (0.650) | 4.01 (0.747) | 3.99 (0.721) |
| *p value* | 0.626 | 0.260 | 0.041* | 0.008** | 0.136 | 0.031* | 0.880 | 0.778 |
| Current Setting of Practice | | | | | | | | |
| Internal medicine | 4.39 (0.646) | 4.15 (0.735) | 4.27 (0.666) | 4.24 (0.655) | 4.16 (0.717) | 4.10 (0.753) | 4.04 (0.787) | 4.39 (0.646) |
| General surgery | 4.39 (0.664) | 4.13 (0.622) | 4.29 (0.535) | 4.22 (0.552) | 4.09 (0.621) | 4.03 (0.738) | 4.07 (0.655) | 4.39 (0.664) |
| Obstetrics, gynecology & pediatrics | 4.31 (0.557) | 4.08 (0.580) | 4.24 (0.550) | 4.19 (0.555) | 4.10 (0.551) | 3.94 (0.719) | 3.97 (0.749) | 4.31 (0.557) |
| Oncology | 4.47 (0.507) | 3.94 (0.814) | 4.18 (0.521) | 4.29 (0.462) | 4.24 (0.554) | 4.00 (0.651) | 3.88 (0.729) | 4.47 (0.507) |
| Emergency | 4.42 (0.499) | 4.24 (0.570) | 4.33 (0.522) | 4.33 (0.564) | 4.18 (0.535) | 3.98 (0.783) | 4.00 (0.853) | 4.42 (0.499) |
| Others | 4.23 (0.712) | 4.00 (0.776) | 4.16 (0.710) | 4.10 (0.677) | 4.08 (0.671) | 3.97 (0.768) | 3.93 (0.749) | 4.23 (0.712) |
| *p value* | 0.107 | 0.002** | 0.009** | 0.050 | 0.004** | 0.437 | 0.115 | 0.140 |

** p*<0.05

*** p*<0.01

**Supplementary material 1**

**The survey questionnaire (English Version)**

| 1. **Place of work** | _____ |
| --- | --- |
| 1. **Contact details (telephone number or email address)** | _____ |
| 1. **Gender** | □Male □Female |
| 1. **Age** | _____ |
| 1. **Current setting of practice** | □Internal medicine □General surgery □Obstetrics, gynecology & pediatrics  □Oncology □Emergency □Others |
| 1. **Length of serving** | _____ |
| 1. **Professional title** | □Junior □Intermediate □Vice-senior □Senior |
| 1. **Executive positions** | □Director of section □Vice director of section □General physician |
| 1. **Highest education** | □Below undergraduate degree □Bachelor’s degree □Master’s degree □Doctoral degree |

| 1. **Frequency and reasons for interactions between you and clinical pharmacists** | | |
| --- | --- | --- |
| 1. Frequency of collaborations: | □Never or rarely □Once a week or more  □Once a day or more | |
| 1. Please indicate the extent of the following statements according to your experience of reasons for collaboration (Reasons for interactions) | □Drug availability queries □Drug alternatives queries \  □Drug dosage queries □Side effects queries  □Drug interactions queries □Other | |
| 1. **Please indicate the extent of the following statements according to your perceptions towards the roles of clinical pharmacists:** | | |
| 1. Providing patient education | □Uncomfortable □Moderately comfortable □Comfortable | |
| 1. Suggesting use of non-prescription medications to patients, e.g. paracetamol | □Uncomfortable □Moderately comfortable □Comfortable | |
| 1. Suggesting use of prescription medications to patients, e.g. antibiotics | □Uncomfortable □Moderately comfortable □Comfortable | |
| 1. Suggesting use of prescription medications to physicians | □Uncomfortable □Moderately comfortable □Comfortable | |
| 1. Treating minor illnesses, e.g. headaches | □Uncomfortable □Moderately comfortable □Comfortable | |
| 1. Designing and monitoring pharmacotherapeutic regimes | □Uncomfortable □Moderately comfortable □Comfortable | |
| 1. Monitoring outcomes of pharmacotherapeutic regimens | □Uncomfortable □Moderately comfortable □Comfortable | |
| 1. Detecting and preventing prescription errors | □Uncomfortable □Moderately comfortable □Comfortable | |
| 1. **Please indicate the extent of the following statements according to your expectations towards the roles of clinical pharmacists:** | | |
| 1. I expect clinical pharmacists to take personal responsibility for resolving any drug-related problems they discover involving patients | | □Strongly disagree □Disagree □Neutral  □Agree □Strongly agree |
| 1. I expect clinical pharmacists to be knowledgeable drug therapy experts | | □Strongly disagree □Disagree □Neutral  □Agree □Strongly agree |
| 1. I expect clinical pharmacists to assist me in designing drug therapy treatment plans for my patients | | □Strongly disagree □Disagree □Neutral  □Agree □Strongly agree |
| 1. I expect clinical pharmacists to educate my patients about the safe and appropriate use of their medication | | □Strongly disagree □Disagree □Neutral  □Agree □Strongly agree |
| 1. I expect clinical pharmacists to monitor my patients’ response to drug therapy and let me know if a patient encounters any drug-related problem | | □Strongly disagree □Disagree □Neutral  □Agree □Strongly agree |
| 1. I expect clinical pharmacists to know the specific indication of each drug I prescribe, even when drugs have more than one approved or recognized indication | | □Strongly disagree □Disagree □Neutral  □Agree □Strongly agree |
| 1. I expect clinical pharmacists to be available to me for consultation when I see patients (e.g. during rounds) | | □Strongly disagree □Disagree □Neutral  □Agree □Strongly agree |
| 1. I expect clinical pharmacists to assist my patients in selecting appropriate non-prescription medications | | □Strongly disagree □Disagree □Neutral  □Agree □Strongly agree |
| 1. **Please indicate the extent of the following statements according to your actual experience with the clinical pharmacists:** | | |
| 1. In my experience, clinical pharmacists are a reliable source of general drug information (e.g., specific facts about drugs which can be found in standard references) | | □Strongly disagree □Disagree □Neutral  □Agree □Strongly agree |
| 1. In my experience, clinical pharmacists are a reliable source of clinical drug information (e.g., information regarding the clinical use of drugs in specific situations) | | □Strongly disagree □Disagree □Neutral  □Agree □Strongly agree |
| 1. Clinical pharmacists routinely counsel my patients regarding the safe and appropriate use of their medications | | □Strongly disagree □Disagree □Neutral  □Agree □Strongly agree |
| 1. Clinical pharmacists routinely inform me if they discover clinical problems with my prescriptions | | □Strongly disagree □Disagree □Neutral  □Agree □Strongly agree |
| 1. Clinical pharmacists routinely inform me about more cost-effective alternatives to the drugs I prescribe | | □Strongly disagree □Disagree □Neutral  □Agree □Strongly agree |
| 1. Clinical pharmacists frequently ask me to clarify for them the drug therapy objectives I have in mind for my patients | | □Strongly disagree □Disagree □Neutral  □Agree □Strongly agree |
| 1. Clinical pharmacists frequently let me know that my patients have experienced some problem with their medication | | □Strongly disagree □Disagree □Neutral  □Agree □Strongly agree |
| 1. In my experience, clinical pharmacists appear willing to take personal responsibility for resolving any drug-related problems they discover | | □Strongly disagree □Disagree □Neutral  □Agree □Strongly agree |

**Supplementary material 2**

**The survey questionnaire (Chinese Version)**

| 1. **您工作医院的全称是？** | _____ |
| --- | --- |
| 1. **您的联系方式（电话号码或电子邮箱）** | _____ |
| 1. **您的性别是？** | □男 □女 |
| 1. **您的年龄是？** | _____岁 |
| 1. **您目前执业的科室是？** | ______ |
| 1. **您在目前岗位或同类岗位的从业年限是？** | _____年 |
| 1. **您的技术职称是？** | □初级职称 □中级职称 □副高级职称 □正高级职称 |
| 1. **您的行政职务是？** | □科室主任 □科室副主任 □普通医生 |
| 1. **您的学历和各学历层次的专业是？** | □本科以下，专业______ □本科，专业______  □硕士，专业______ □博士，专业______ |

| 1. **您和临床药师合作的情况是：** | | |
| --- | --- | --- |
| 1. 我与临床药师合作的频率 | □从不/极少 □至少一周一次 □一日一次或以上 | |
| 1. 我在何种情況下会与临床药师合作（多选） | □药物可及性查询 □药物替代查询 □药物剂量查询  □药物副作用查询 □药物相互作用查询 □其它 | |
| 1. **临床药师的下列工作让您感到舒适（即有助于您完成工作）的程度是**： | | |
| 1. 指导患者用药 | □舒适 □较为舒适 □不舒适 | |
| 1. 向患者提供非处方建议，如建议患者使用扑热息痛 | □舒适 □较为舒适 □不舒适 | |
| 1. 向患者提供处方建议，如建议患者使用抗生素 | □舒适 □较为舒适 □不舒适 | |
| 1. 向医生提供处方建议 | □舒适 □较为舒适 □不舒适 | |
| 1. 治疗轻微的疾病，如治疗患者头疼 | □舒适 □较为舒适 □不舒适 | |
| 1. 设计及监测药物治疗方案 | □舒适 □较为舒适 □不舒适 | |
| 1. 监测药物治疗方案的结果 | □舒适 □较为舒适 □不舒适 | |
| 1. 检查及消除处方中的错误 | □舒适 □较为舒适 □不舒适 | |
| 1. **您对临床药师有下列期望**： | | |
| 1. 我希望临床药师能负责发现并解决病人的全部药物治疗相关问题。 | | □非常不同意 □不同意 □不确定 □同意 □非常同意 |
| 1. 我希望临床药师具有丰富的药物治疗知识。 | | □非常不同意 □不同意 □不确定 □同意 □非常同意 |
| 1. 我希望临床药师协助我为患者设计药物治疗方案。 | | □非常不同意 □不同意 □不确定 □同意 □非常同意 |
| 1. 我希望临床药师指导患者安全和适当地使用药物。 | | □非常不同意 □不同意 □不确定 □同意 □非常同意 |
| 1. 我希望临床药师监测患者接受药物治疗的反应，并让我知道患者是否遇到任何与药物治疗有关的问题。 | | □非常不同意 □不同意 □不确定 □同意 □非常同意 |
| 1. 我希望临床药师知道我开的每种药物的具体适应症，即使药物有一种以上的获批或被认可的适应症。 | | □非常不同意 □不同意 □不确定 □同意 □非常同意 |
| 1. 当我检查患者时（如查房期间），我希望临床药师为我提供咨询。 | | □非常不同意 □不同意 □不确定 □同意 □非常同意 |
| 1. 我希望临床药师帮助患者选择合适的非处方药。 | | □非常不同意 □不同意 □不确定 □同意 □非常同意 |
| 1. **您对与您合作的临床药师有下列评价：** | | |
| 1. 根据我的经验，临床药师是药物基本信息的可靠来源（如临床药师了解药品说明书等标准资料内药物的具体事实信息）。 | | □非常不同意 □不同意 □不确定 □同意 □非常同意 |
| 1. 根据我的经验，临床药师是药物临床信息的可靠来源（如临床药师了解文献资料中报告的特定情形下的药品临床使用信息）。 | | □非常不同意 □不同意 □不确定 □同意 □非常同意 |
| 1. 临床药师经常向患者提供安全用药、合理用药的咨询。 | | □非常不同意 □不同意 □不确定 □同意 □非常同意 |
| 1. 临床药师在日常工作中会告知我处方存在的临床治疗问题。 | | □非常不同意 □不同意 □不确定 □同意 □非常同意 |
| 1. 临床药师在日常工作中会告诉我，我开的药有更具成本效益的替代药品。 | | □非常不同意 □不同意 □不确定 □同意 □非常同意 |
| 1. 临床药师在日常工作中会请我阐明患者的药物治疗目标。 | | □非常不同意 □不同意 □不确定 □同意 □非常同意 |
| 1. 临床药师经常让我了解到患者用药时遇到的一些药物治疗相关问题。 | | □非常不同意 □不同意 □不确定 □同意 □非常同意 |
| 1. 根据我的经验，临床药师似乎愿意在解决他们所发现的药物相关问题时，承担个人责任。 | | □非常不同意 □不同意 □不确定 □同意 □非常同意 |
